# High Expression of Protein Tyrosine Kinase 7 Significantly Associates with Invasiveness and Poor Prognosis in Intrahepatic Cholangiocarcinoma

**DOI:** 10.1371/journal.pone.0090247

**Published:** 2014-02-28

**Authors:** Jing Jin, Han Suk Ryu, Kyoung Bun Lee, Ja-June Jang

**Affiliations:** Department of Pathology, Seoul National University College of Medicine, Seoul, Korea; Yonsei University College of Medicine, Republic of Korea

## Abstract

**Background:**

The incidence, prevalence, and mortality of intrahepatic cholangiocarcinoma (ICC) are increasing worldwide. Protein tyrosine kinase-7 (PTK7) is upregulated in many common human cancers. However, its expression in ICC has not been studied. The present study aimed to explore the underlying mechanism of PTK7 in ICC.

**Materials and Methods:**

The role of PTK7 was studied in vitro by suppressing PTK7 expression in ICC cell lines. The in vivo effect of PTK7 was evaluated using a nude mouse model inoculated with a human ICC cell line. We also examined the role of PTK7 in human ICC samples.

**Results:**

Cells with high PTK7 expression exhibited higher proliferation, DNA synthesis, invasion, and migration abilities than did cells with low PTK7 expression. The knockdown of PTK7 with small interfering RNA (siRNA) in high PTK7 expressing cells resulted in impairment of invasion, migration, and DNA synthesis through the regulation of several cell-cycle-related proteins. It also induced cell apoptosis and decreased phospho-RhoA expression. In a xenograft nude mouse model, PTK7 siRNA resulted in a reduction of the tumor size, compared with scrambled siRNA injection. PTK7 expression was higher in human ICC than in the normal bile duct. Patients with low expression of PTK7 had a longer disease-free survival and overall survival than those with high expression.

**Conclusions:**

PTK7 expression plays an important role in the invasiveness of ICC cells and leads to a poor prognosis in ICC patients. Thus, PTK7 can be used as a prognostic indicator, and the inhibition of PTK7 expression could be a new therapeutic target for ICC.

## Introduction

Intrahepatic Cholangiocarcinoma (ICC) may arise through the malignant transformation of cholangiocytes in any part of the biliary tree. Biliary epithelial cells undergo genetic and epigenetic alterations in various regulatory genes, which accumulate and lead to the activation of oncogenes and the dysregulation of tumor suppressor genes, generating irreversible changes in the physiology of the cholangiocytes [1].

The high mortality and poor outcome of this disease are attributed to the lack of available tools for its early diagnosis and treatment. Surgery represents the only curative treatment for ICC, however, surgery is only feasible at an early stage and is characterized by a high rate of recurrence [2]. Recent therapeutic options include brachytherapy and photodynamic therapy, although their effects have not yet been established.

Protein tyrosine kinase-7 (PTK7) is a relatively new and less-studied member of the receptor tyrosine kinase superfamily. It was originally identified as a gene expressed in a colon cancer-derived cell line, but it is not expressed in human adult colon tissues [3]. PTK7 expression is upregulated in many common human cancers, including colon cancer, lung cancer, gastric cancer, and acute myeloid leukemia [3]–[8].

Recently, PTK7 was identified as a novel regulator of non-canonical Wnt or planar cell polarity (PCP) signaling [9]. These PCP signaling pathways control cellular polarity, cell mobility, and signal, resulting in a modification of the cytoskeleton [10].

Previously, we have found that PTK7 was associated with a poor prognosis in patients with intrahepatic cholangiocarcinoma using cDNA mediated annealing, selection, extension and ligation CHiP study (unpublished data).

The aim of this study was to explore the role of PTK7 in ICC. To our knowledge, this is the first insight into the role of PTK7 in ICC and the underlying mechanism of its involvement in ICC both *in vitro* and *in vivo*.

## Materials and Methods

### Ethics Statement

The paraffin-embedded tissues surgically resected from the patients were used as a retrospective study after its use for diagnosis. The tissue samples were released from the diagnostic archive, and did not identify the patients. The need for written informed consent was waived by the Institutional Review Board of Seoul National University Hospital. The present study was conducted in accordance with the ethical standards of the Helsinki Declaration in 1975, after approval of the Institutional Review Board of Seoul National University Hospital (H-1011-046-339).

The animal experiment was approved by the Seoul National University Hospital Institutional Animal Care and Use Committee (SNUH-IACUC No: 13-0051). All surgery was performed under anesthesia, and all efforts were made to minimize suffering.

### Cell lines and cell culture

The cholangiocarcinoma cell lines, JCK, SCK, Choi-CK, and Cho-CK were kindly provided by the Division of Gastroenterology and Hepatology of Chonbuk National University Hospital (Jeonju, Republic of Korea) [11]. HuCCT1 and OZ cell lines were purchased from the Japanese Collection of Research Bioresources Cell Bank. The HepG2 cell line was used as a positive control for PTK7 expression. The HuCCT1, OZ, and HepG2 cells were maintained in RPMI 1640 medium, William's E medium, and Eagle's minimal essential medium, respectively. The JCK, SCK, Choi-CK, and Cho-CK cell lines were all maintained in Dulbecco's Modified Eagle Medium. Each medium was supplemented with 10% fetal bovine serum and 1% penicillin-streptomycin.

### Small interfering RNA (siRNA) and transfection

Transfection of three PTK7-specific siRNA and scrambled negative siRNA (Integrated DNA Technologies, Iowa, USA) at a final concentration of 60 nM was performed using G-fectin transfection reagent (Genolution Pharmaceuticals, Seoul, Korea) (Table 1). Cells were maintained under normal culture conditions and were harvested for analysis at 72 hours after transfection.

**Table 1:** List of small interfering RNA (siRNA) and polymerase chain reaction sequences used in this study.

Sequence name	Forward	Reverse
Scrambled siRNA	5′CUU CCU CUC UUU CUC UCC CUU GUG A3′	3′UCA CAA GGG AGA GAA AGA GAG GAA GGA5′
#1 PTK7 siRNA	5′GCC ACA GCA CAA GUG AUA AGA UGC A3′	3′UGC GGU GUC GUG UUC ACU AUU CUA CGU5′
#2 PTK7 siRNA	5′GGC AUG UCU UCA AUC UCU GCU AGG UGA3′	3′CCG UAC AGA AGU UAG AGA CGA UCC A5′
#3 PTK7 siRNA	5′ACA ACC GCU UUG UGC AUA AGG ACT T3′	3′GUU GUU GGC GAA ACA CGU AUU CCU GAA5′
GAPDH	5′AAG GTC GGA GTC AAC GGA TTT GGT3′	3′TAC TGG TGT CAG GTA CGG TAG TGA5′
PTK7	5′TCT GAT GGT CAG AGC AAC CAC ACA3′	3′TAA ACT CGG TGC CCA ATG TCG5′

### Cell proliferation and DNA synthesis ability analysis

Cells were seeded into a 96-well plate for 18 hours. Next, cell viability was evaluated using a MTT cell proliferation kit (Roche, Mannheim, Germany) and DNA synthesis was detected using an ELISA bromodeoxyuridine (BrdU) colorimetric kit (Roche, Mannheim, Germany).

### Western Blotting

Protein samples were boiled, loaded onto sodium dodecyl sulfate gels and electrotransferred onto polyvinylidene difluoride membranes (Millipore Corporation, MA, USA) [12]. The membranes were incubated with appropriate primary and HRP-conjugated secondary antibodies (Zymed, CA, USA) for the required times.

### Reverse-Transcription Polymerase Chain Reaction

Reverse-Transcription Polymerase Chain Reaction (RT-PCR) of PTK7 and GAPDH was performed as described [13]. The PCR primer and probe sets are listed in Table 1.

### Annexin V staining and FACS analysis

After incubation with or without siRNA-PTK7 for 72 hours, an apoptosis kit (Medical&Biological Laboratories Co., Nagoya, Japan) was used to assay for siRNA-induced apoptosis. A volume of 5 µL of Annexin V-FITC and 2.5 µL of propidium iodine were added before analyzing using a BD FACS caliber flow cytometer (BD Biosciences, MA, USA).

### Invasion assay

The invasiveness of cells was assayed using transwell membranes coated with 500 ng/μL of Matrigel (BD Biosciences, San Jose, CA, USA) [14]. Penetrated cells were counted in 5 microscopic fields by using a digital camera with a 400× objective lens (Olympus, Tokyo, Japan).

### Wound healing assay

Cells were seeded into 96-well plates. After 2–4 days, each culture plate reached a confluent cell monolayer. A small area was disrupted by scratching a line through the cell layer with a 200-μL pipette tip. Photographs were taken with a digital camera at 0, 12, 24, 36, and 48 hours after scratching.

### Xenograft nude mouse model

Ten male BALB/C nude mice, 7 weeks old and 20–25 g in weight, were inoculated subcutaneously with HuCCT1 cells (1×10^7^) into the dorsal area. After 5 weeks when the tumor volume reached 180 mm3 on average, nude mice were randomly divided into 2 groups (five mice in each group). PTK7 siRNA (40 µmol/L) dissolved in 200 µL of AteloGene® (KOKEN, Tokyo, Japan) was administered directly into the tumour every 3 days for three times. A scrambled siRNA was used as control. Tumors were removed 10 days after the final siRNA treatment and were fixed in formalin and embedded in paraffin blocks for further study. Tumor volume was assessed with the formula: 0.4×length x width x height. Apoptosis was quantified by using the terminal deoxyribonucleotidyl transferase-mediated dUTP nick end labeling (TUNEL) assay. The tumor cell proliferation rate was analyzed by Ki67 staining.

### Selection of patients and tissue specimens

Samples of ICCs were collected from 194 patients who had undergone surgical resection at Seoul National University Hospital in Seoul, from 1992 to 2010. We divided the patients into test set (all the 78 cases of patients in 1992–2001) and validation set (the 116 cases of patients in 2002–2010).The hematoxylin and eosin (H&E) stained pathological slides and clinicopathological medical records of all the cases were reviewed. Follow-up periods ranged from 1 to 196 months (median follow-up duration: 30.0 months). The patients' age at the time of diagnosis ranged from 37 to 80 years (median age: 61.5 years). Tumor size ranged from 0.3 to 26.0 cm (mean tumor size ± SD: 5.53±0.25 cm). Disease-free survival (DFS) was defined as the time to local or distant progression. Overall survival (OS) was defined as the time to ICC-related death. All 194 patients had no evidence of postoperative residual malignancy. Fifty-one of the patients examined, had received adjuvant chemotherapy. With regard to the underlying liver disease, 15 patients had chronic hepatitis, 12 of whom had hepatitis B virus infection and 3 had hepatitis C virus infection; 3 patients had clonorchis sinensis; and 3 patients had hepatolithiasis. Tumor differentiation was categorized based on the grading system described by the World Health Organization classification [15]. To use as controls, normal bile duct tissues were collected from patients with hepatolithiasis, who had undergone surgical resection.

### Construction of tissue microarray

Suitable areas with two representative tumor areas for each case were marked on the H&E stained sections, and core tissue specimens (2 mm in diameter) were collected from individual paraffin-embedded tissues and rearranged in new tissue array blocks by using a trephine apparatus (SuperBioChips Laboratories, Seoul, Korea).

### Immunohistochemical staining and evaluation of clinical samples

The β-catenin staining was considered positive if both a loss of membrane staining and an aberrant expression of cytoplasmic and/or nuclear staining were detected. PTK7, Ki67, and TUNEL expression was evaluated as defined in a previous study [16] using Aperio ImageScope (Aperio Technologies, CA, USA).

The positivity percentage of PTK7 was calculated using the average of positive intensities divided by the total numbers of stained pixels. All cases were scored using a histological scoring (HSCORE) method. Specimens with a HSCORE>60 were regarded as PTK7 positive, whereas those with a HSCORE≤60 were regarded as PTK7 negative [17].

### Statistical analysis

*In vitro* data and clinical results were compared using the Student's t-test. Significance of *in vivo* data was assessed by Mann-Whitney test. DFS and OS were calculated by the Kaplan-Meier method and compared with the log-rank test. The Cox proportional-hazard regression model was used to explore the effects of the clinicopathologic variables and PTK7 expression on survival. The results were considered to be statistically significant when the *P* values≤0.05. All tests were performed using the SPSS 17.0 software (SPSS, Chicago, IL, USA).

## Results

### Different expression of PTK7 in six cholangiocarcinoma cell lines

**Figure 1:**
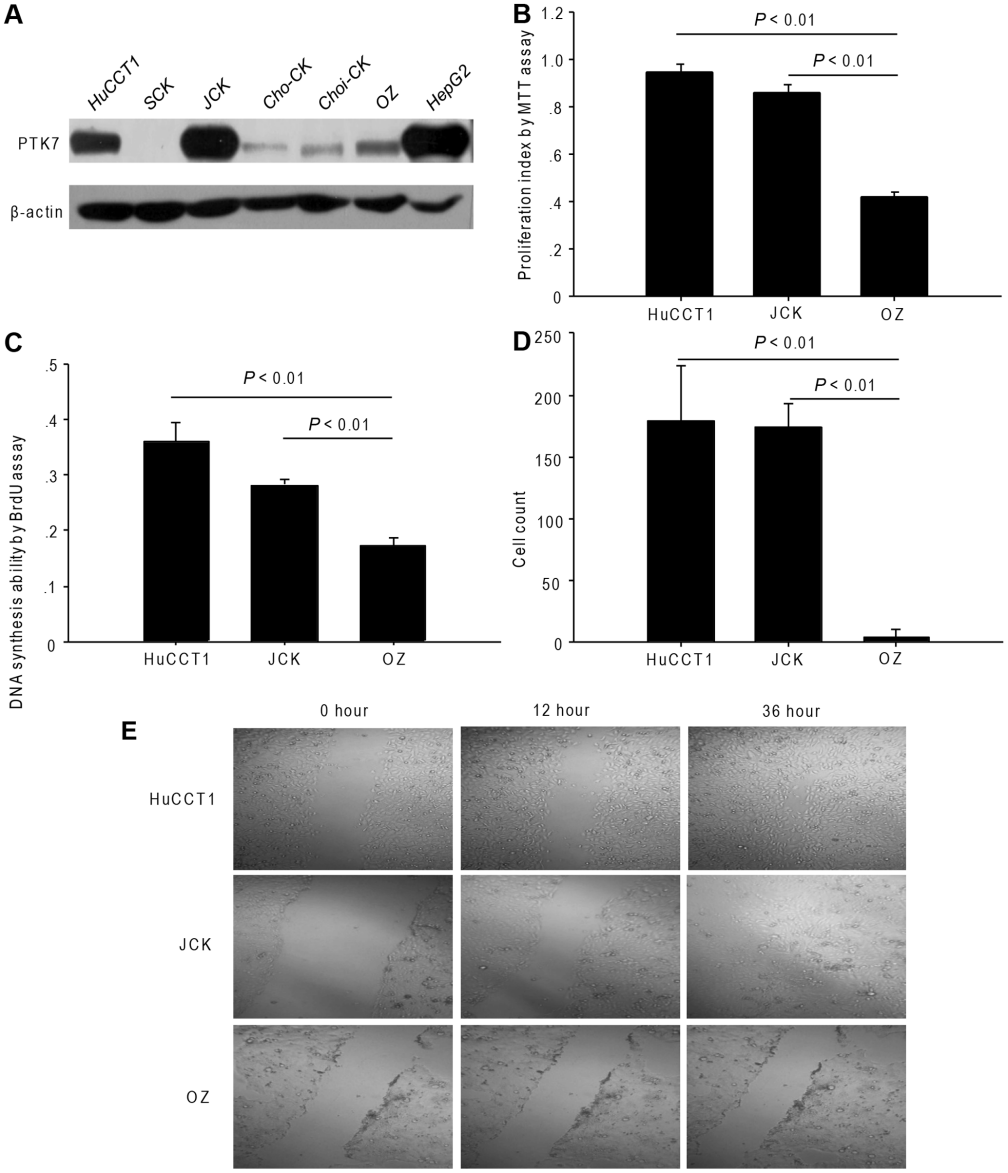
Different characteristics of cholangiocarcinoma cells lines. (A) PTK7 expression in six cholangiocarcinoma cell lines. (B) Proliferation ability, (C) DNA synthesis ability and (D) invasion ability of the HuCCT1, JCK and OZ cell lines. (E) Migration ability assessed by the migration assay (original magnification, 100×). *P*<0.01, comparing HuCCT1 or JCK cells with OZ cells. All experiments were replicated thrice with triplicate repeated measures within each replication for each time point. Data represent the mean ± SD.

Firstly, six human cholangiocarcinoma cell lines (HuCCT1, SCK, JCK, Cho-CK, Choi-CK, and OZ) were tested with the PTK7 antibody. The PTK7 were strongly expressed in HuCCT1 and JCK cells, while weakly expressed in SCK, Cho-CK, Choi-CK, and OZ cells (Figure 1A). We further excluded out the Choi-CK cell line because it was a hilar type cholangiocarcinoma cell line. During the cell culture, the SCK and Cho-CK cell lines were slightly changing their original morphologies, so we also excluded these 2 cell lines out of our further experiment.

### Proliferation, DNA synthesis, invasion, and migration abilities are higher in HuCCT1 and JCK cells than in OZ cells

Considering that HuCCT1 and JCK cells show higher expression levels of PTK7 than OZ cells, we assumed that the different behavior was according to their different PTK7 expression levels. We found that the HuCCT1 and JCK cells proliferated faster than OZ cells (Figure 1B, *P*<0.01). DNA synthesis rate was also higher in HuCCT1 and JCK cells (Figure 1C, *P*<0.01). Additionally, the invasion and migration abilities of HuCCT1 and JCK cells were stronger than those of OZ cells (Figure 1D and 1E, *P*<0.01).

### PTK7-specific siRNA successfully knocks down the PTK7 expression in HuCCT1 and JCK cells

**Figure 2:**
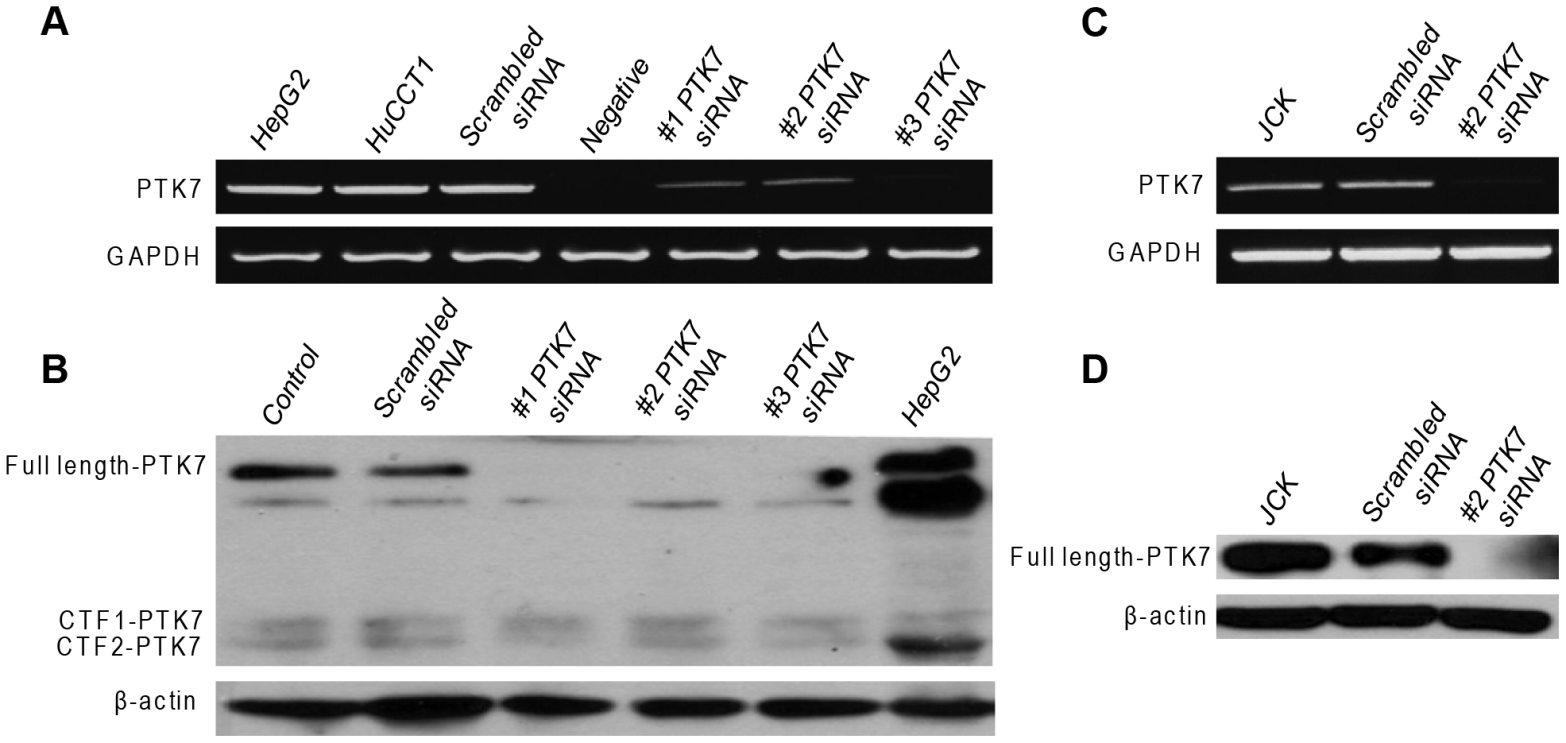
PTK7-specific siRNA silencing in HuCCT1 and JCK cells. (A) PTK7 mRNA and (B) protein levels after transfection of PTK7 siRNAs in HuCCT1 cells. (C) PTK7 mRNA and (D) protein levels after transfection of PTK7 siRNAs in JCK cells.

Following transfection with the PTK7-specific siRNAs, the expression of PTK7 mRNA (Figure 2A and 2C) and protein (Figure 2B and 2D) was successfully suppressed in both cell lines. On the contrary, PTK7 siRNA did not change CTF1 or CTF2 expression levels in HuCCT1 cells (Figure 2B).

### PTK7-specific siRNA treatment decreases the invasion and migration abilities and impairs DNA synthesis ability in HuCCT1 and JCK cells

**Figure 3:**
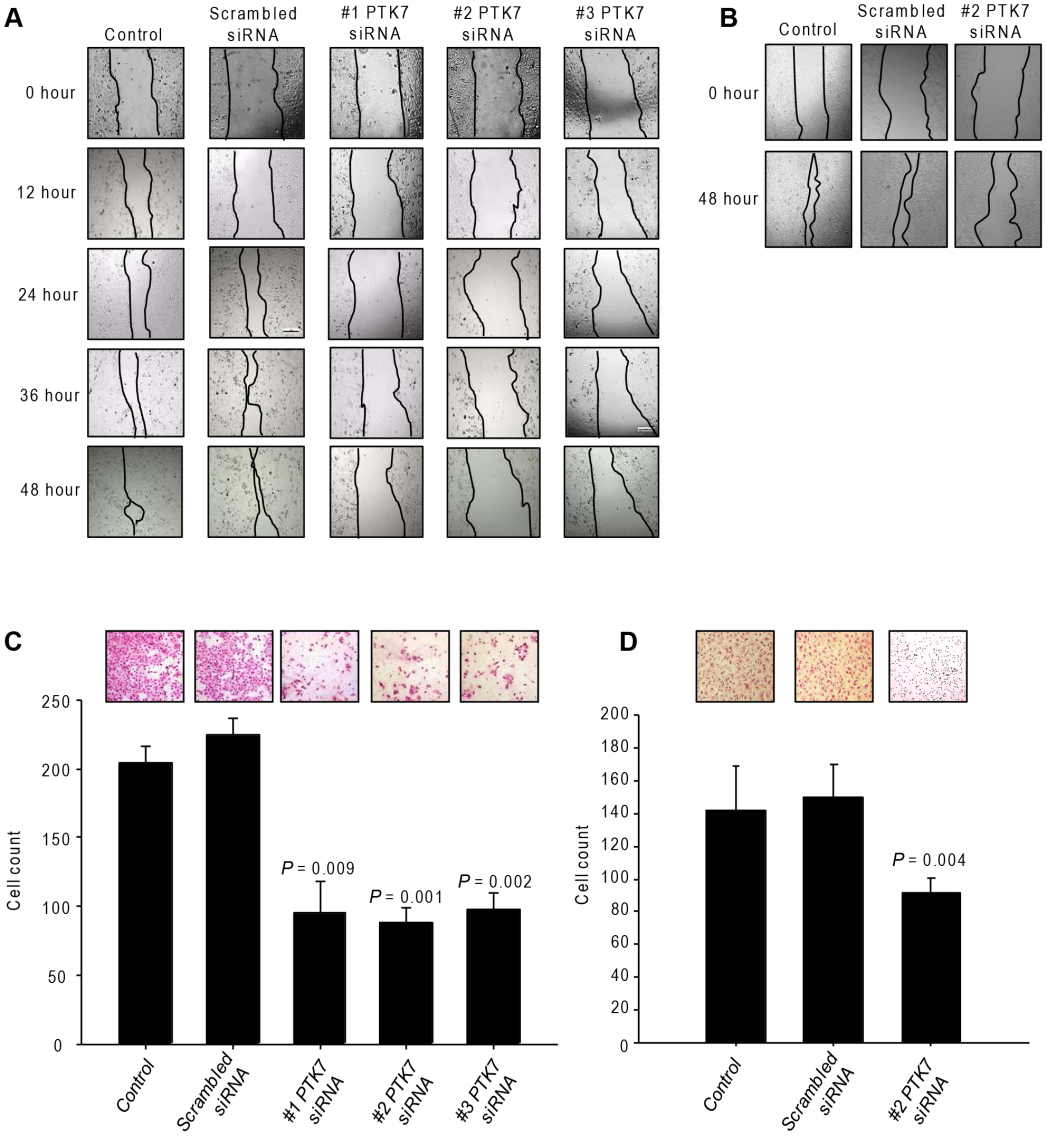
Effect of PTK7-specific siRNA on HuCCT1 and JCK cells. (A) Migration and (C) invasion ability with siRNA treatment in HuCCT1 cells. (B) Migration and (D) invasion ability with siRNA treatment in JCK cells. All experiments were replicated thrice with triplicate repeated measures within each replication for each time point.

Compared with the scrambled siRNA-treated group, the knockdown of PTK7 decreased the cell mobility into the wound (Figure 3A). The cell population that migrated through the Matrigel-coated transwell was higher than that in the scrambled siRNA group (Figure 3C). The same results were seen in JCK cells (Figure 3B and 3D).

### Effect of PTK7 silencing on cell-cycle-related proteins in HuCCT1 cells

**Figure 4:**
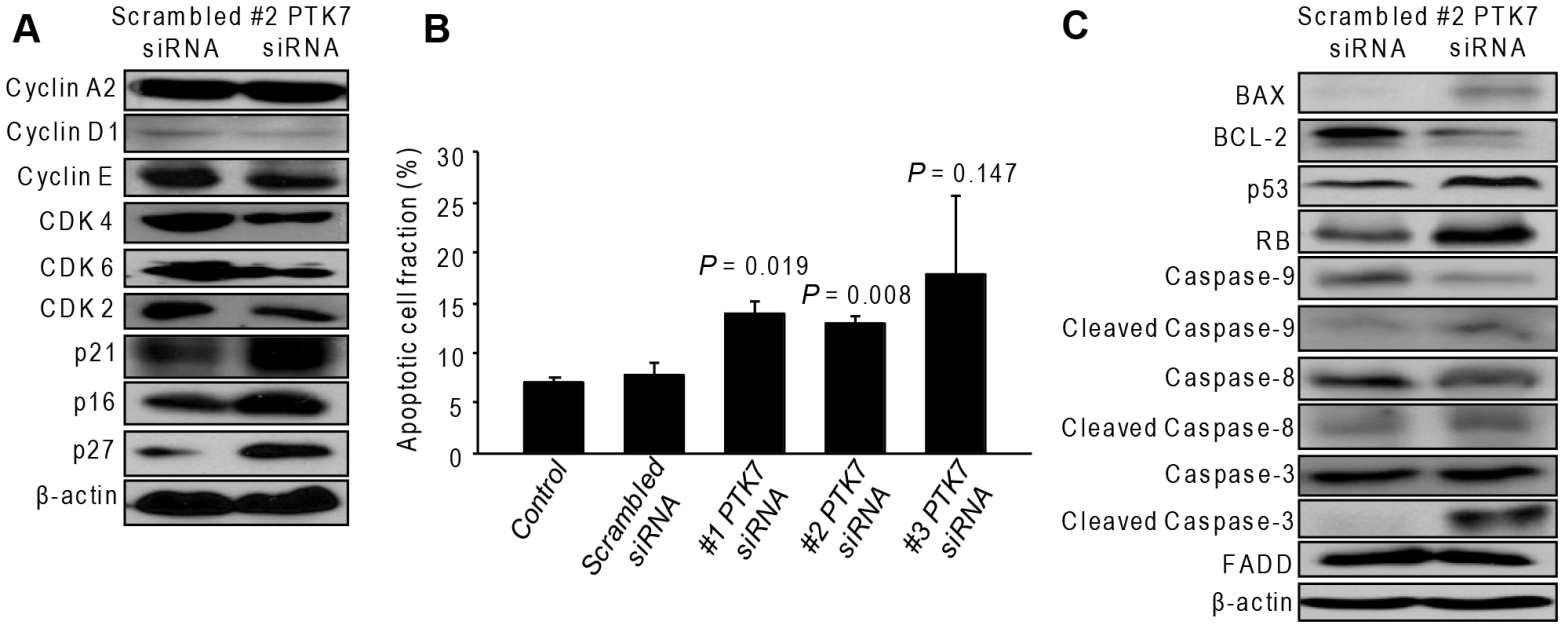
Effect of PTK7-specific siRNA on cell cycle and apoptosis in HuCCT1 cells. (A) Cell-cycle-related protein expressions with siRNA treatment. (B) The percentage of apoptotic cells with siRNA treatment. (C) Effect of siRNA on the apoptosis-related proteins. *P* values are presented for comparison with scrambled siRNA group. All experiments were replicated thrice with triplicate repeated measures within each replication for each time point. Data represent the mean ± SD.

Since the HuCCT1 cells and JCK cells were presenting the same characteristics, we only used the HuCCT1 cells for further mechanism studies. We detected several proteins related to the cell cycle process. Cell-cycle-related proteins such as cyclin A2 and cyclin E were not affected. However, Cdk2, Cdk4, Cdk6, and cyclin D1 levels were slightly decreased, whereas p16, p21, and p27 levels were increased by PTK7 silencing (Figure 4A).

### PTK7 silencing induces cell apoptosis in HuCCT1 cells

Cell apoptosis was induced by PTK7-specific siRNA transfection (Figure 4B). In addition, BAX the tumour suppressor genes p53 and RB were increased, followed by a decrease of BCL-2. Moreover, the apoptotic cascade was activated by the PTK7-specific siRNA, with an increase in the levels of cleaved caspase-3 and caspase-9. However, caspase-8 and Fas-associated death domain (FADD) were not affected (Figure 4C).

### Effect of PTK7 silencing on the planar cell polarity signaling pathway in HuCCT1 cells

**Figure 5:**
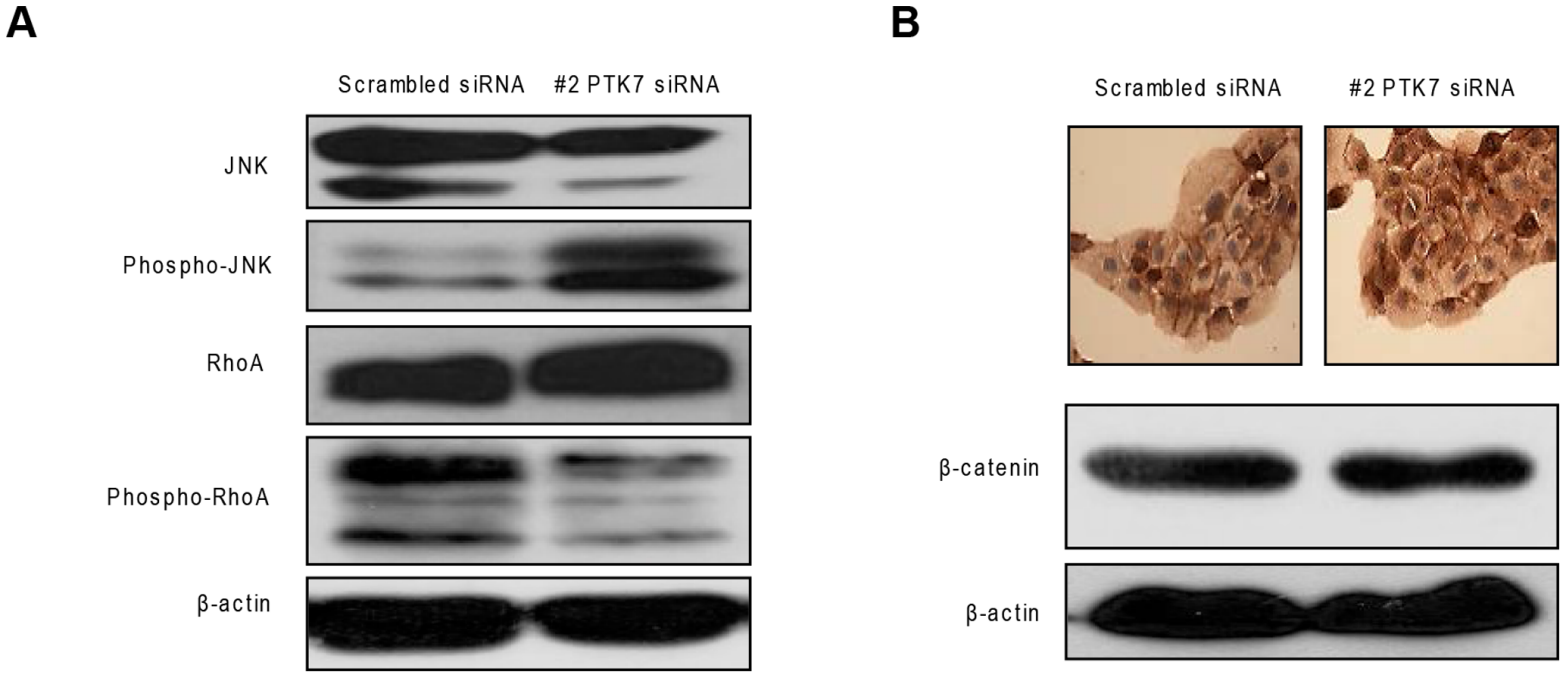
Effect of PTK7-specific siRNA on Wnt pathway in HuCCT1 cells. (A) Results of the PTK7 silencing on the non-canonical Wnt pathway (PCP)-related proteins. (B) Western blotting (lower panel) and immunohistochemistry staining (upper panel) of β-catenin on HuCCT1 cells with siRNA treatment (original magnification, 400×).

We considered that the PTK7-dependent abilities of invasion and migration are associated with the PCP pathway, which activates phosphor-RhoA and JNK, and leads to cytoskeleton reorganization of the cell membrane. When cells were transfected with PTK7-specific siRNA, JNK phosphorylation increased together with a decrease in the phospho-RhoA level (Figure 5A). Besides the non-canonical Wnt/β-catenin pathway, there is also the canonical Wnt/β-catenin pathway. The data showed that PTK7 does not influence the canonical Wnt/β-catenin pathway (Figure 5B, lower panel), which was further confirmed by the immunohistochemical staining of β-catenin, showing no nuclear translocation (Figure 5B, upper panel).

### PTK7 silencing reduced tumor formation in xenograft nude mouse model

**Figure 6:**
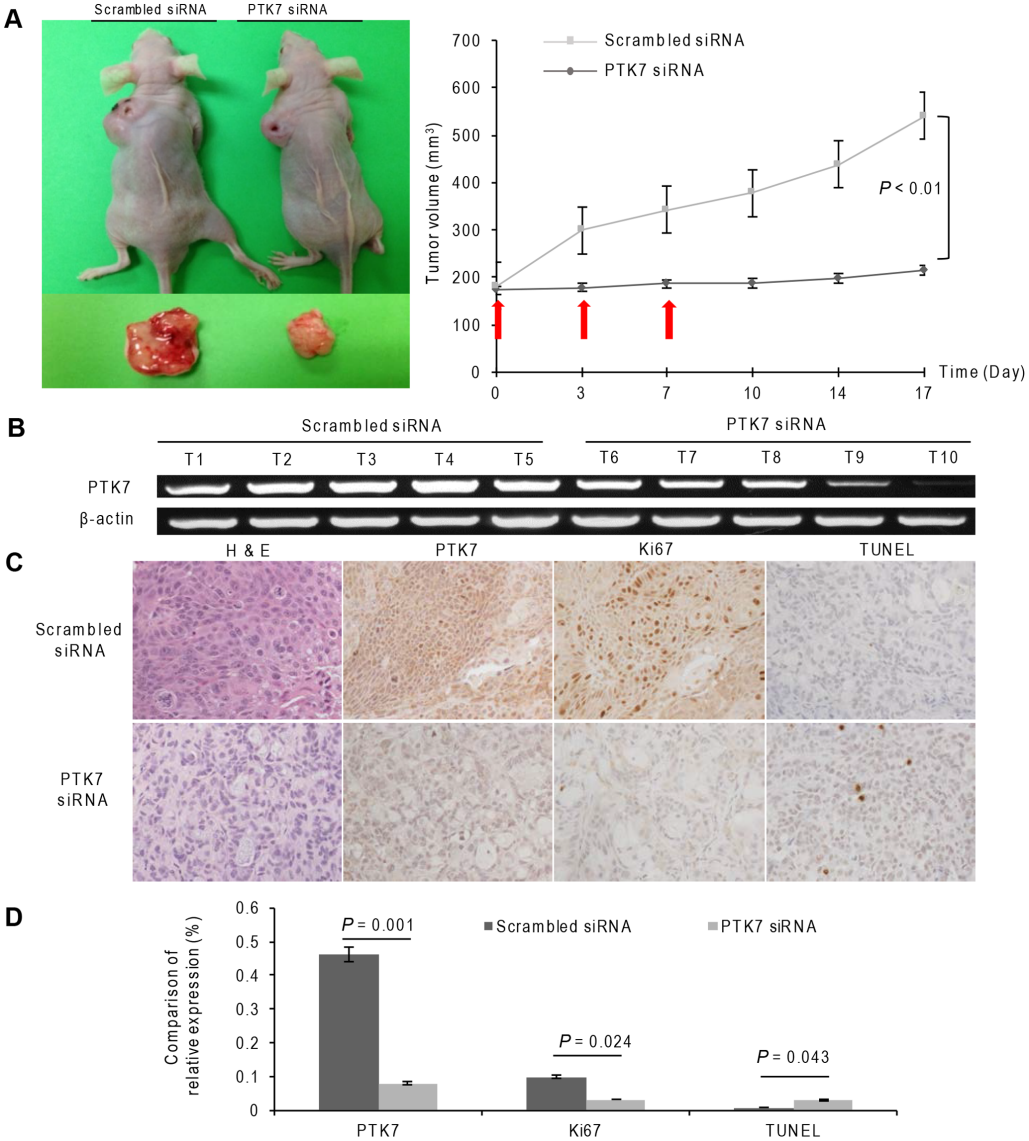
Effect of PTK7 silencing in a xenograft nude mouse model. (A) Representative nude mice in the PTK7-specific siRNA group and scrambled siRNA group (left panel). Tumor volume of the PTK7-specific siRNA-treated and the scrambled group (right panel). (B) PTK7 mRNA expression in each tumor. (C) Representative H&E, PTK7, Ki67, and TUNEL staining in the PTK7-specific siRNA and scrambled siRNA treated groups (original magnification, 200×). (D) Comparison of relative PTK7, Ki67, and TUNEL staining. *P* values are presented for comparison with the scrambled siRNA group. Data represent the mean ± SD.

To examine the possible activity of PTK7-specific siRNA on tumorigenesis in vivo, a xenograft nude mouse model was used. Mean tumor volumes in PTK7-specific siRNA-treated mice were reduced in comparison with those of the control mice (Figure 6A, left panel). Silencing of PTK7 dramatically suppressed tumor formation in the xenografts of the nude mice (Figure 6A, right panel, *P*<0.01). Figure 6B showed the PTK7 were successfully silenced by PTK7-specific siRNA. Tumor sections of the xenografts were analyzed by hematoxylin and eosin staining followed by TUNEL and Ki67 staining (Figure 6C). The group treated with the PTK7 siRNA tended to have more TUNEL positive and less Ki67 positive cells than the scrambled siRNA group (Figure 6D).

### PTK7 was strongly expressed in human ICC than normal bile duct

**Figure 7:**
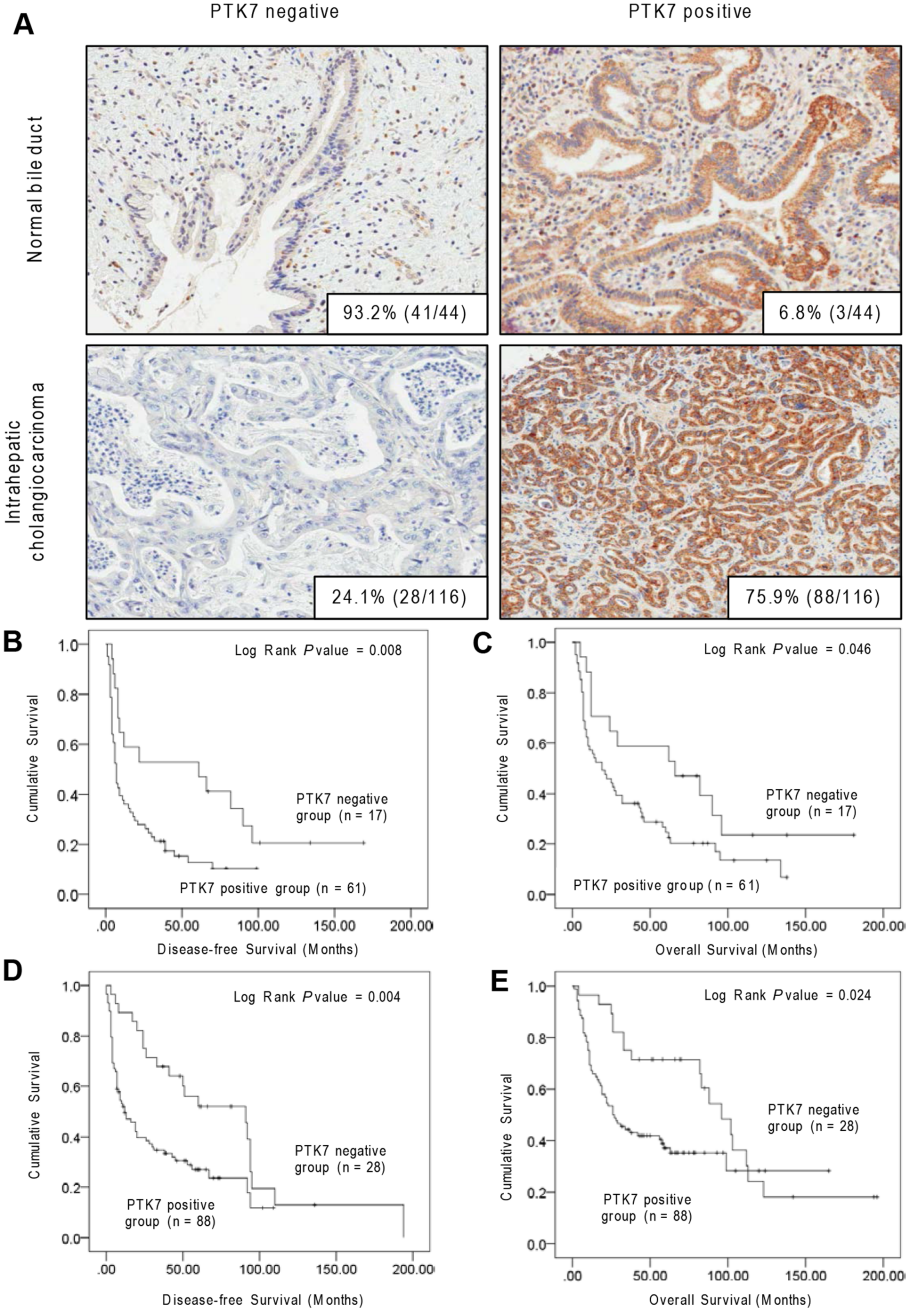
PTK7 expression in human ICC and normal bile duct tissue. (A) PTK7 immunohistochemistry staining for ICC and normal bile duct tissue. In normal bile duct, 93.2% (41/44) of cases stained negatively for PTK7 (upper left), 6.8% (3/44) of cases stained positively for PTK7 (upper right). In ICC, 24.1% (28/116) of cases stained negatively for PTK7 (lower left), 75.9% (88/116) of cases stained positively for PTK7 (lower right) (original magnification, 200×). Kaplan–Meier curves for (B) DFS and (C) OS of patients with positive or negative PTK7 expression in test set, and (D) DFS and (E) OS of validation cohort.

According to the results of TMA-based immunohistochemistry, PTK7 was mainly expressed in the cytoplasm. The positive rates of PTK7 in the cytoplasm were 75.9% (88/116) and 6.8% (3/44) in the ICC and normal bile duct, respectively (Figure 7A, *P*<0.01).

### PTK7 protein expression for predicting patient outcome

Kaplan–Meier univariate survival analysis revealed that PTK7 overexpression was associated with a poor disease-free survival (DFS) (Figure 7B, *P* = 0.008) and overall survival (OS) (Figure 7C, *P* = 0.046). Multivariate Cox proportional hazards regression analysis revealed that patients with a high PTK7 expression had a 2.3-fold greater risk of disease recurrence and a 1.8-fold greater risk of disease-related death (*P* = 0.015 and 0.036, respectively, Table 2). In this model, tumor size and angiolymphatic invasion were also identified as potential predictors of DFS and OS. The similar results were confirmed in the validation set (Figure 7D and 7E; Table 2). Different PTK7 expressions showed no significances among the clinicopathological variables (Table S1).

**Table 2:** Independent predictive factors of disease-free survival (DFS) and overall survival (OS) by multivariate analysis (Cox proportional hazards model).

Variables	Test set				Validation set			
	DFS		OS		DFS		OS	
	HR (95% CI)	*P* value	HR (95% CI)	*P* value	HR (95% CI)	*P value*	HR (95% CI)	*P value*
PTK7 expression								
Negative vs Positive	2.319 (1.175–4.580)	***0.015***	1.801 (1.040–3.118)	***0.036***	3.304 (1.749–6.244)	***<0.001***	2.498 (1.298–4.807)	***0.006***
Tumor size (cm)								
≤5 vs >5	1.875 (1.102–3.191)	***0.021***	1.832 (1.055–3.179)	***0.031***	1.496 (1.277–1.889)	***0.018***	2.081 (1.166–3.713)	***0.013***
Neural invasion								
Absent vs Present	1.249 (0.631–2.473)	*0.208*	1.649 (0.821–3.310)	*0.16*	1.039 (0.631–1.710)	*0.881*	1.396 (0.834–2.339)	*0.205*
Angiolymphatic invasion								
Absent vs Present	2.04 (1.200–3.470)	***0.008***	1.809 (1.038–3.155)	***0.037***	2.246 (1.190–4.241)	***0.013***	1.870 (0.999–3.497)	***0.050***
IHM								
Absent vs Present	0.543 (0.243–1.209)	*0.135*	0.800 (0.351–1.826)	*0.597*	1.384 (0.856–2.239)	*0.185*	1.436 (0.858–2.402)	*0.168*
EHE								
Absent vs Present	0.982 (0.568–1.698)	*0.947*	0.738 (0.414–1.315)	*0.302*	0.721 (0.423–1.229)	*0.229*	1.389 (0.840–2.295)	*0.200*
pT stage (7th AJCC)								
T1 & T2 vs T3 & T4	2.319 (0.756–3.475)	*0.523*	1.216 (0.626–2.360)	*0.563*	1.769 (1.027–3.047)	***0.040***	1.855 (1.057–3.258)	***0.031***

Notes: HR, hazard ratio; CI, confidential interval; DFS, disease-free survival; OS, overall survival; IHM, intrahepatic metastasis; EHE, extrahepatic extension. All experiments were replicated thrice with triplicate repeated measures within each replication for each time point.

## Discussion

Our present study found that a high PTK7 expression contributed to the proliferation, invasion, and migration abilities of ICC cells, through the PCP signaling pathway. PTK7 was highly expressed in the tissue samples of human ICC but not in normal bile duct samples.

Cellular proliferation can be suppressed either by interruption of the cell cycle or by cell apoptosis. Firstly, we investigated the cell-cycle-related proteins. A previous study proved that the silencing of PTK7 can lead to the inhibition of cell proliferation and apoptosis in colon cancer cells [18]. In this study, we first demonstrated that silencing of PTK7 slightly decreased Cdk2, Cdk4, Cdk6, and cyclin D1 and increased p16, p21, and p27 expression.

Two distinct but convergent pathways, the extrinsic and intrinsic, can initiate apoptosis. Our results showed that silencing of PTK7 did not have an effect on FADD and cleaved caspase-8, suggesting no effect in the extrinsic apoptotic pathway. In contrast, pro-apoptotic BAX was increased by PTK7 silencing, followed by a decrease of anti-apoptotic BCL-2. The apoptotic cascade was also activated by PTK7-specific siRNA, with an increase of cleaved caspase-3 and caspase-9. These results demonstrated that PTK7 silencing leads to apoptosis in HuCCT1 cells via the intrinsic mitochondrial pathway. Our data showed that PTK7-specific siRNA increases p21 levels. In addition to growth arrest p21, which was discovered via a senescent cell-derived inhibitor, can mediate cellular senescence. Thus, p21 here serves not only as a cell proliferation inhibitor but also as an apoptosis initiator. In addition, the tumor suppressor genes p53 and RB were also increased by the knockdown of PTK7.

This is the first time that cell-cycle-related proteins and tumor suppressor genes have been studied in a PTK7-dependent manner in ICC cell lines. We also found that PTK7-specific siRNA significantly decreased the abilities of invasion and migration in HuCCT1 cells. The majority of deaths from carcinoma are caused by secondary growths that result from tumor invasion and metastasis. Recently, PTK7 was identified as a novel regulator of the non-canonical Wnt or PCP signaling pathway [9]. Since embryonic processes, which are pivotally related to PCP signaling, share many similarities with cancer development, it is noteworthy to investigate further its role in ICC.

The non-canonical PCP signaling is activated by ligands such as Wnt5a or Wnt11. Signaling is transduced by the Frizzled receptor and the adaptor protein Dishevelled and activates the RhoA and Rac GTPases and their respective targets, Rho-associated kinase and JNK. The functional assays of Peradziryi et al. [19] showed that PTK7 activates the non-canonical Wnt signaling and inhibits the canonical Wnt signaling. We found that β-catenin was localized in the cell membrane, regardless of whether a PTK7-specific siRNA was present, which implies that PTK7 is not involved in the canonical Wnt signaling, thereby confirming Peradziryi's finding [19].

There has been debate about the role of JNK in the Wnt/PCP signaling pathway. Some researchers have proposed that JNK serves as a downstream event of RhoA and is involved in the cytoskeleton rearrangement of Wnt/PCP [20], [21]. However, recent studies have suggested that JNK activation in Wnt/PCP has pro-apoptotic action rather than changing the cytoskeleton structure [22]. In the present study, phospho-JNK expression was increased by PTK7 silencing. In addition to the result that PTK7-specific siRNA can induce cell apoptosis, we hypothesized that the action may be partially related to JNK activation, since the role of the JNK in apoptosis is both cell-type- and stimulus-dependent. In addition, the role of JNK in apoptosis depends on the activity of other cellular signaling pathways [23].

RhoA was initially considered to be involved in the regulation of the actin cytoskeleton [24]. The RhoA/ROCK pathway regulates numerous endothelial cellular functions such as migration and adhesion [25]. We found that PTK7 silencing impaired the migration and invasion abilities with a downregulation of activated phospho-RhoA, which is in agreement with the results from other studies.

Previously, Na et al. had reported that a soluble100-kDa fragment of PTK7 inhibits the tube formation, migration, and invasion of endothelial cells and angiogenesis [13]. However, the shedding of PTK7 is cell-type-dependent and has not been observed in cholangiocytes so far. The PTK7 fragments would diffuse out into the extracellular space without a significant concentration in cancer tissues. In contrast, PTK7-CTF2 is able to be effectively concentrated in the nucleus, and thus activate signaling pathways that promote tumorigenesis and metastasis. In this study, we found that PTK7-specific siRNA did not affect the intracellular cleavage of PTK7-CTF2, but the full length of PTK7 was knocked down. As a result, the migration and invasion abilities of the ICC cells were inhibited, providing evidence that the intact PTK7 molecule is oncogenic in the HuCCT1 cell line. The animal experiment confirmed the role of PTK7 in ICC tumorigenesis.

Finally, we assessed the PTK7 expression in surgically resected ICC specimens. As expected, PTK7 was highly expressed in ICC but not in normal bile duct tissue. The overexpression of PTK7 was associated with poor DFS and poor OS.

This is the first report of the functional role of PTK7 in ICC. Our results show that high PTK7 expression may play an important role in ICC cell invasion and lead to a poor prognosis. Thus, PTK7 can be used as a prognostic indicator and the inhibition of PTK7 expression could be a new therapeutic target for ICC.

## Supporting Information

Table S1 **Clinicopathological variables and PTK7 expression in intrahepatic cholangiocarcinoma (test set, n = 78; validation set, n = 116).**
(DOCX)
