# Supplementary material for: High Expression of Protein Tyrosine Kinase 7 Significantly Associates with Invasiveness and Poor Prognosis in Intrahepatic Cholangiocarcinoma
Source: PLoS One. 2014 Feb 28;9(2):e90247. doi: 10.1371/journal.pone.0090247 (PMC3938661; doi:10.1371/journal.pone.0090247)
Supplement: Table S1 — Clinicopathological variables and PTK7 expression in intrahepatic cholangiocarcinoma (test set, n = 78; validation set, n = 116). (DOCX) [file pone.0090247.s001.docx]

**Table S1. Clinicopathological variables and PTK7 expression in intrahepatic cholangiocarcinoma (test set, n = 78; validation set, n = 116)**

| Variables | Test set | | | | Validation set | | | |
| --- | --- | --- | --- | --- | --- | --- | --- | --- |
|  | PTK7 expression | | | | PTK7 expression | | | |
|  | Total | Positive | Negative | *P* | Total | Positive | Negative | *P* |
|  | (n = 78) | (n = 61) | (n = 17) |  | (n =116) | (n = 88) | (n = 28) |  |
| Sex |  |  |  |  |  |  |  |  |
| Male | n = 61 | 47 (77.0%) | 14 (23.0%) | *0.639* | n = 88 | 63 (71.6%) | 25 (28.4%) | ***0.042*** |
| Female | n = 17 | 14 (82.4%) | 3 (17.6%) |  | n = 28 | 25 (89.3%) | 3 (10.7%) |  |
| Age (years) |  |  |  |  |  |  |  |  |
| ≤ 60 | n = 35 | 26 (74.3%) | 9 (25.7%) | *0.449* | n = 56 | 37 (66.1%) | 19 (33.9%) | ***0.017*** |
| > 60 | n = 43 | 35 (81.4%) | 8 (18.6%) |  | n= 60 | 51 (85.0%) | 9 (15.0%) |  |
| Size (cm) |  |  |  |  |  |  |  |  |
| ≤ 5 | n = 46 | 32 (69.6%) | 14 (30.4%) | ***0.027*** | n = 65 | 48 (73.8%) | 17 (26.2%) | *0.567* |
| > 5 | n = 32 | 29 (90.6%) | 3 (9.4%) |  | n = 51 | 40 (78.4%) | 11 (21.6%) |  |
| Multiplicity |  |  |  |  |  |  |  |  |
| Present | n = 5 | 5 (100.0%) | 0 (0.0%) | *0.222* | n = 5 | 5 (100.0%) | 0 (0.0%) | *0.197* |
| Absent | n = 73 | 56 (76.7%) | 17 (23.3%) |  | n = 111 | 83 (74.8%) | 28 (25.2%) |  |
| EHE |  |  |  |  |  |  |  |  |
| Present | n = 36 | 31 (86.1%) | 5 (13.9%) | *0.117* | n = 59 | 45 (76.3%) | 14 (23.7%) | *0.917* |
| Absent | n = 42 | 30 (71.4%) | 12 (28.6%) |  | n = 57 | 43 (75.4%) | 14 (24.6%) |  |
| pT stage (7th AJCC) |  |  |  |  |  |  |  |  |
| T1 & T2 | n = 61 | 47 (77.0%) | 14 (23.0%) | *0.639* | n = 86 | 67 (77.9%) | 19 (22.1%) | *0.384* |
| T3 & T4 | n = 17 | 14 (82.4%) | 3 (17.6%) |  | n = 30 | 21 (70.0%) | 9 (30.0%) |  |
| Angiolymphatic invasion |  |  |  |  |  |  |  |  |
| Present | n = 33 | 26 (78.8%) | 7 (21.2%) | *0.915* | n = 57 | 43 (75.4%) | 14 (24.6%) | *0.917* |
| Absent | n = 45 | 35 (77.8%) | 10 (22.2%) |  | n = 59 | 45 (76.3%) | 14 (23.7%) |  |
| Neural invasion |  |  |  |  |  |  |  |  |
| Present | n = 14 | 10 (71.4%) | 4 (28.6%) | *0.498* | n = 35 | 26 (74.3%) | 9 (25.7%) | *0.794* |
| Absent | n = 64 | 51 (79.7%) | 13 (20.3%) |  | n = 81 | 62 (76.5%) | 19 (23.5%) |  |
| IHM |  |  |  |  |  |  |  |  |
| Present | n = 11 | 10 (90.9%) | 1 (9.1%) | *0.271* | n = 17 | 13 (76.5%) | 4 (23.5%) | *0.948* |
| Absent | n = 67 | 51 (76.1%) | 16 (23.9%) |  | n = 99 | 75 (75.8%) | 24 (24.2%) |  |

EHE, extrahepatic extension; pT, pathological T stage; IHM, intra hepatic metastasis.
